# Total Reconstruction of the Upper Lip Using Bilateral Nasolabial Flaps, Submental Flap, and Mucosa Graft following Complete Resection for Squamous Cell Carcinoma

**DOI:** 10.1155/2015/782151

**Published:** 2015-11-26

**Authors:** O. G. Oseni, A. E. Fadare, M. O. Majaro, P. B. Olaitan

**Affiliations:** Department of Surgery, Ladoke Akintola University of Technology Teaching Hospital, Osogbo, Osun 230105, Nigeria

## Abstract

Lip reconstruction following resection for tumour or following extensive trauma may pose a challenge. This is more so when the resection is total and a complete lip has to be constructed. We present a case of lip reconstruction following a total resection of the upper lip. The procedure used in this case was a combination of bilateral nasolabial flaps with a submental flap and buccal mucosal graft lining. We believe that this provides an alternative method of total upper lip reconstruction with minimal disruption of the facial aesthesis.

## 1. Introduction

The perioral area is a focal point of both spoken and nonspoken communication and an area of great aesthetic importance. Its mobile nature and varied contours make it a reconstructive challenge. The lips are a focus of facial beauty, and their central location does not permit concealment of unsightly scars or asymmetrical results. Both lips, but especially the lower lip, are at risk for cutaneous malignancy because of their prominent location [1]. The reconstruction of composite defects of the upper lip following oncological resection is challenging from both functional and aesthetic perspectives [2]. Structural restoration of the lip must take into consideration internal lining and external skin cover as well as the functional goals of oral competence, the ability to eat and drink, and intelligible speech. Aesthetically, there are numerous subtle, but very important, variations in contour, color, and texture. With no underlying bony or cartilaginous framework, they are extremely sensitive to distortion [3].

We present a case of complete reconstruction of the upper lip following resection for extensive squamous cell carcinoma with bilateral nasolabial flaps, submental flap in addition to mucosa graft for lining. We belief this should be added to the options of treating such a large defect especially where microvascular procedures are not feasible.

## 2. Case Report

A. G was a 56-year-old farmer who presented with extensive upper lip ulcer of about 3-year duration. Ulcer on the upper lip had started with a small nodular swelling on the upper lip which subsequently extended to involve the whole of the upper lip. There was associated purulent discharge with offensive odour which caused a social embarrassment to the man. He had attempted suicide but was counseled and later brought to the hospital. There was no previous burn injury or scar on the lip prior to the swelling.

**Figure 1:**
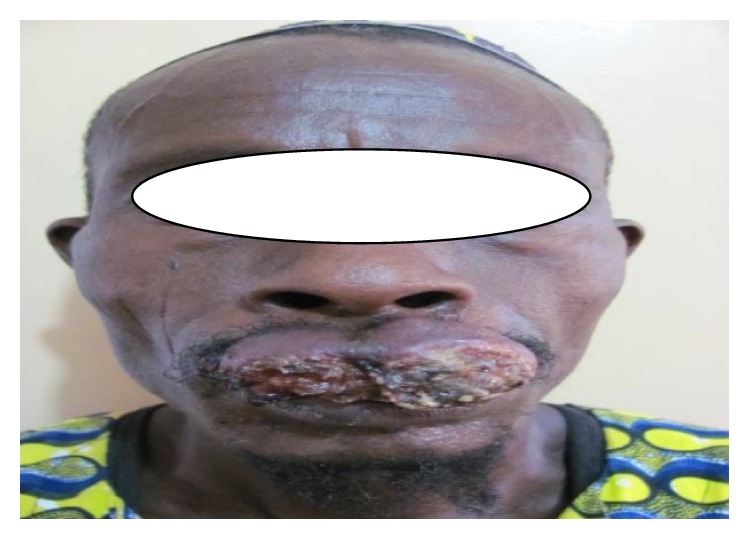
Extensive exophytic ulcer on the upper lip.

The main finding on examination was an extensive exophytic ulcer on the whole of the upper lip extending almost to the commissures bilaterally and just about 1 cm short of the root of the columella (Figure 1).

**Figure 2:**
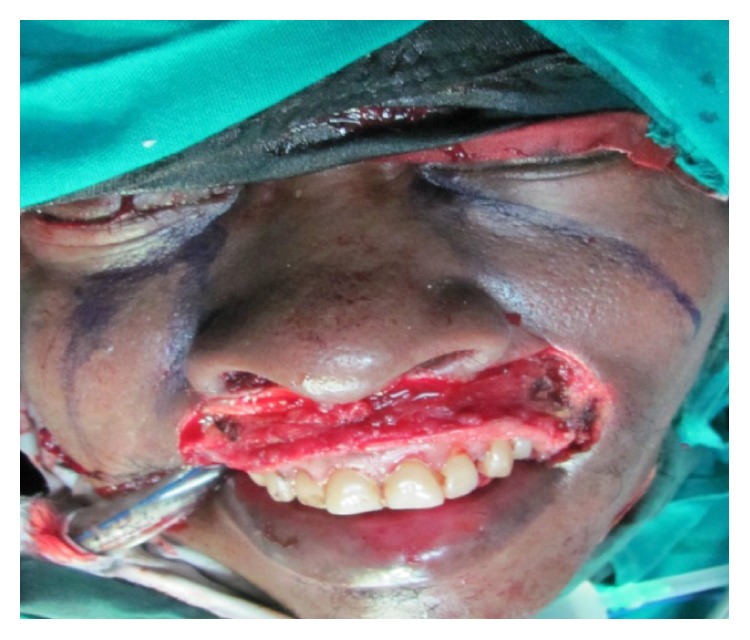
Postexcision of the tumor and marking for nasolabial flaps.

He had a total resection of the lesion with a complete resection of the upper lip laterally on either side to the commissures and up to the root of the columela superiorly (Figure 2).

**Figure 3:**
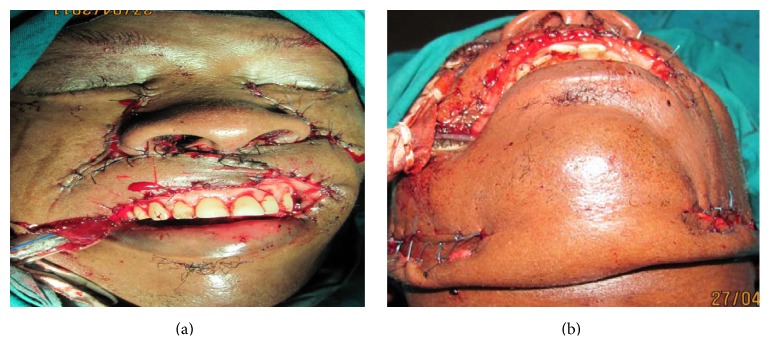
Insertion of bilateral nasolabial flaps and elevation of bipedicled mental flap.

**Figure 4:**
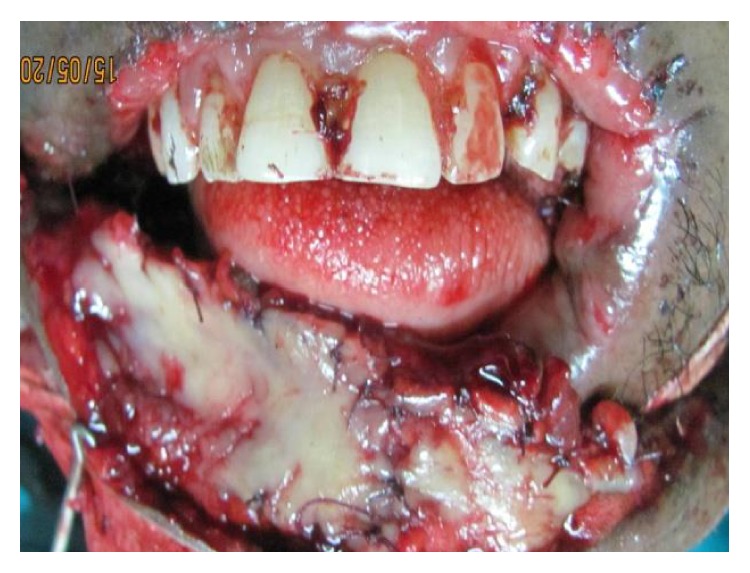
Submental flap lined with mucosal graft.

Bilateral inferiorly based nasolabial flaps were raised and brought to interdigitate with each other over the exposed teeth (Figure 3(a)). A bipedicled submental flap was raised with a 2.5 cm pedicle from either side of the cheeks with the origin of the flap from the areas of the mastoid bones (Figure 3(b)). The submental part of the flap was not raised until about two weeks later (Figure 4).

At the second stage of the surgery, the nasolabial flaps were observed to have done well and about 1.0 cm inferior margin of the flaps was deepithelialised to accept the submental flap. A bipedicled submental flap of about 3.5 cm wide by 3.5 cm was raised and the inferior 2.5 cm portion of the flaps lined with mucosa obtained from the cheek (Figure 4).

The flap was then sutured onto the deepithelialised portion of the nasolabial flaps, thus allowing some free margin of the lip that is not adherent with the soft tissue over the teeth of the upper lip.

The pedicles were divided on the fourth and eight days, respectively, following the second stage of the procedure.

**Figure 5:**
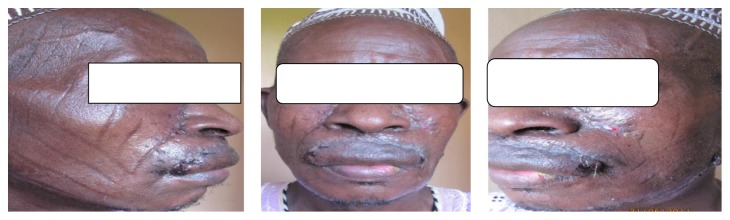
Postoperative picture views.

**Figure 6:**
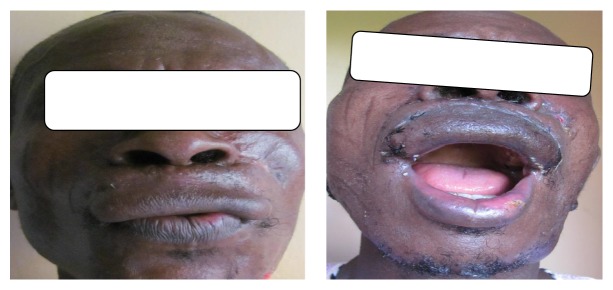
Postoperative picture: whistling and mouth opening.

He had topical application of 5-fluorouracil postoperatively. Patient was quite satisfied with the result with good aesthetic and functional outcome (Figures 5 and 6).

## 3. Discussion

The perioral area is a focal point of both spoken and nonspoken communication and an area of great aesthetic importance. Its mobile nature and varied contours make it a reconstructive challenge. Surgical resection of the lip for malignancies brings a full-thickness defect of lip tissues, and management of the resulting lip defect needs a surgical technique that maximizes functional and cosmetic outcomes [4].

Although the use of local tissue flaps forms the basic concept of lip reconstruction, these are often limited when an extensive part of the lip is to be reconstructed. Coupled with this is the need to minimize facial defects and scarring especially in the black patients who are prone to severe hypertophic scars and keloids.

Full-thickness lip repair poses some challenges. This challenge is more pronounced when a total reconstruction is contemplated with minimal distortion of the structures of the face. Aesthetic considerations also dictate that the reconstruction should attempt to minimize or at least camouflage the extent of disfigurement resulting from such extensive surgery. Few published reports have addressed the issues related to upper lip reconstruction [5]. Upper lip defects less than one-fourth of total upper lip length are typically closed directly, but larger defects require reconstruction.

The combined dorsalis pedis cutaneous, extensor hallucis, and digitorum brevis muscle conjoined free flap is useful for a moderate or subtotal defect of the full-thickness lip when local or regional flaps are not applicable [6]. Full-thickness reconstruction of the subtotal upper lip defect and the restoration of the function and appearance of the columella has been done using modified bilateral nasolabial flaps [7]. The use of the bilateral nasolabial flaps with bipedicled submental flap and cheek mucosa is an addition to the options for consideration where a total upper lip reconstruction is needed with an advantage of no microvascular involvement. This means that the procedure is possible with any surgeon wherever he is with minimal facilities. This method could reconstruct anatomical features and function of the lip precisely.

Facial scarring is also reduced as scar from the submental flap is well hidden and the flap can provide hair on the upper lip, thereby masking the reconstruction and absence of the philtral dimple as well as other philtral prominences like the cupid bow and the columns. This is therefore an option when considering reconstruction of total upper lip loss.
